# *C*¹ Positive Surface over Positive Scattered Data Sites

**DOI:** 10.1371/journal.pone.0120658

**Published:** 2015-06-09

**Authors:** Farheen Ibraheem, Malik Zawwar Hussain, Akhlaq Ahmad Bhatti

**Affiliations:** 1 National University of Computer and Emerging Science, Lahore, Pakistan; 2 Department of Mathematics, University of the Punjab, Lahore, Pakistan; Tianjin University of Technology, CHINA

## Abstract

The aim of this paper is to develop a local positivity preserving scheme when the data amassed from different sources is positioned at sparse points. The proposed algorithm first triangulates the irregular data using Delauny triangulation method, therewith interpolates each boundary and radial curve of the triangle by *C*¹ rational trigonometric cubic function. Half of the parameters in the description of the interpolant are constrained to keep up the positive shape of data while the remaining half are set free for users’ requirement. Orthogonality of trigonometric function assures much smoother surface as compared to polynomial functions. The proposed scheme can be of great use in areas of surface reconstruction and deformation, signal processing, CAD/CAM design, solving differential equations, and image restoration.

## 1. Introduction

Data measured or amassed from many engineering and scientific fields, is often positioned at sparse points. For example, meteorological measurements at different weather stations [1], density measurements on different positions within the human body, heart potential measurements at random points in the diagnosis of various ailments of heart [2], 3D photography, aeronautical engineering and industrial design, structural graph networks [3], graph entropy [4], [5], [6]. A visual model is often required to get a clear understanding of underlying phe- nomena as colossal amount of data is difficult to analyse or communicate a message in raw form. Further, a meticulous visual representation obligates the interpolating function to affirm intrinsic attributes of data like positivity, monotonicity and convexity. Although, tensor product provides a robust medium for fitting surface to rectilinear data sites, it can not be used to fit a surface over sparse data points. This paper addresses the problem of retaining positivity over scattered data points.

Several approaches have been proposed in literature to address the problem of positivity preserving interpolating surfaces. Amidor [7] surveyed method to interpolate scattered data necessitating from electronic imaging system. The author mainly examined radial basis function method, tetrahedral interpolation, cubic triangular interpolation, triangle based blending interpolation, inverse distance method and neutral neighbourhood. The difference between scattered data interpolation and scattered data fitting was also demonstrated in the survey. Cubic and quintic Hermite interpolants were used for preserving monotonicity, positivity and convexity of discrete data by [8]. Piah, Goodman, Unsworth [9] first triangulated the data points by Delaunay triangulation and constructed the interpolating surface consisting of “cubic Bezier triangular patches”. Positivity of data was achieved by imposing sufficient conditions on Bezier ordinates in each triangular patch. The proposed scheme was local and *C* ^1^ continous. Hussain and Hussain [10] arranged the scattered data over a triangular grid to preserve the positivity and monotonicity. The authors used a cubic interpolant with one parameter to interpolate the boundary of each triangular patch while linear interpolant was used in Nielson side vertex method to obtain radial curves. Final surface patch was obtained by convex combination of interpolants. Positivity and monotonicity was retained by deriving data dependent constraints on free parameters. *C* ^1^ Quadratic splines and Powell-Sabin splines were used as interpolating function to tackle the problem of range restricted univariate and bivariate scattered data by Hermann et. al. [11]. The authors obtained a system of inequalities for the gradients and positivity was accomplished by deriving sufficient conditions on this system. A *C* ^1^ local rational cubic Bernstein Bezier interpolatory scheme was proposed by Hussain and Hussain [12] to retain positivity of scattered data. In each triangular patch, inner and boundary Bezier ordinates were confined for positivity. If in any triangular grid, Bezier ordinates failed to attain positive shape of data, then these were varied by the weights described in formation of rational cubic Bernstein Bezier interpolant. Sarfraz et. al. [1] established a local *C* ^1^ approach to keep up the positivity of scattered data positioned over a triangular domain. They employed *C* ^1^ rational cubic function with four parameters in Nielson side vertex technique to formulate the interpolating surface. Two of the four parameters were constrained for positivity.

Although several approaches have been proposed to retain the positivity of data, little attention has been paid towards the use of trigonometric basis function. This paper develops a positivity preserving scheme for scatter data by taking *C* ^1^ rational trigonometric function [13] into account. Delaunay triangulation method has been used to place scatter data as vertices of triangle. Nielson side vertex method [14] has been employed in each triangle to construct triangular patches. The *C* ^1^ rational trigonometric cubic function [13] with four parameter has been used for the interpolation along boundary and radial curve of the triangle. Positivity is attained by deriving data dependent condition on half of the parameters in the description of *C* ^1^ rational trigonometric cubic function [13].

The remainder of the paper is formulated as: Section 2 reviews the ratonal trigonometric cubic function [13]. Nielson side vertex method [14] to formulate triangular patches is detailed in Section 3. Positivity preserving algorithm is developed and explained in Section 4. Section 5 demonstrates the developed algorithm and presents graphical results. Section 5 summarizes this research and draws conclusion.

## 2. Rational Trigonometric Cubic Function

Let {(*x*_*i*_, *y*_*i*_), *i* = 0,1,2, …, *n*−1} be the given set of data points defined over the interval [*a*, *b*] where *a* = *x*_0_ < *x*_1_ < *x*_2_ < … < *x*_*n*_ = *b*. A piecewise rational trigonometric cubic function is defined over each subinterval *I*_*i*_ = [*x*_*i*_, *x*_*i*+1_] as

$$\begin{array}{c} S_{i}\left(x\right)=\frac{p_{i}\left(\theta \right)}{q_{i}\left(\theta \right)} \end{array}$$ (1)

$$\begin{array}{ccc} p_{i}\left(\theta \right) & = & \alpha_{i}f_{i}{\left(1-\sin\theta \right)}^{3}+\left\{\beta_{i}f_{i}+\frac{2h_{i}\alpha_{i}d_{i}}{\pi }\right\}\sin\theta {\left(1-\sin\theta \right)}^{2}\\ & + & \left\{\gamma_{i}f_{i+1}-\frac{2h_{i}\delta_{i}d_{i+1}}{\pi }\right\}\cos\theta {\left(1-\cos\theta \right)}^{2}+\delta_{i}f_{i+1}{\left(1-\cos\theta \right)}^{3}\\ q_{i}\left(\theta \right) & = & \alpha_{i}{\left(1-\sin\theta \right)}^{3}+\beta_{i}\sin\theta {\left(1-\sin\theta \right)}^{2}+\gamma_{i}\cos\theta {\left(1-\cos\theta \right)}^{2}+\delta_{i}{\left(1-\cos\theta \right)}^{3} \end{array}$$

where

$$\begin{array}{c} \theta =\frac{\pi }{2}\left(\frac{x-x_{i}}{h_{i}}\right),h_{i}=x_{i+1}-x_{i},\;i=0,1,2,...,n-1 \end{array}$$

The rational trigonometric cubic function (Eq 1) satisfy the following properties:

$$\begin{array}{c} S\left(x_{i}\right)=f_{i},S\left(x_{i+1}\right)=f_{i+1},S^{\prime }\left(x_{i}\right)=d_{i},S^{\prime }\left(x_{i+1}\right)=d_{i+1}. \end{array}$$ (2)

*d*_*i*_ and *d*_*i*+1_ are derivative at the endpoints of the interval *I*_*i*_ = [*x*_*i*_, *x*_*i*+1_]. *α*_*i*_, *β*_*i*_, *γ*_*i*_ and *δ*_*i*_ are the free parameters. The following result has been proved in [13].

**Theorem 2.1** *The C* ^1^ *piecewise rational trigonometric cubic function preserve the positivity of positive data if in each subinterval I_i_ = [x_i_,x_i+1_], the parameters β_i_ and γ_i_ satisfy the following sufficient conditions*

$$\begin{array}{c} \beta_{i}=u_{i}+\max\left\{0,\frac{-2h_{i}d_{i}\alpha_{i}}{\pi f_{i}}\right\},u_{i}>0,\\ \gamma_{i}=v_{i}+\max\left\{0,\frac{2h_{i}d_{i+1}\delta_{i}}{\pi f_{i+1}}\right\},v_{i}>0. \end{array}$$

## 3. Nielson Side Vertex Method

Consider a triangle △*V*_1_ *V*_2_ *V*_3_ with vertices *V*_1_, *V*_2_, *V*_3_ having edges *e*_1_, *e*_2_, *e*_3_ and *u*, *v*, *w* be the barycentric coordinates such that any point *V* on the triangle can be written as:

$$\begin{array}{c} V≐uV_{1}+vV_{2}+wV_{3}, \end{array}$$ (3)

where

$$\begin{array}{c} u+v+w=1\;\text{and}\;u,v,w\geq 0. \end{array}$$

The interpolant defined by Nielson [14] to generate surface over each triangular patch is defined as the following convex combination:

$$\begin{array}{c} P\left(a,b,c\right)=\frac{v^{2}w^{2}Q_{1}+u^{2}w^{2}Q_{2}+u^{2}v^{2}Q_{3}}{v^{2}w^{2}+u^{2}w^{2}+u^{2}v^{2}}. \end{array}$$ (4)

where ${Q_{i}}^{\prime }s$ represent line segments joining vertices $V_{i}^{\prime }s$ to points $S_{i}^{\prime }s$ on the opposite boundary. Eq (4) interpolates data at the vertices as well as first order derivatives at the boundary. Since the barycentric coordinates at the vertices of triangle is simultaneously zero, the interpolant Eq (4) takes the following values:

$$\begin{array}{c} P(a,b,c)=Q_{1}\;\text{when}\;v=w=0,\\ P(a,b,c)=Q_{2}\;\text{when}\;u=w=0,\\ P(a,b,c)=Q_{3},\text{when}\;v=u=0, \end{array}$$

where *Q*_*i*_, *i* = 1,2,3 are the ordinate values at the vertices *V*_*i*_, *i* = 1,2,3 of triangle.

## 4. Positive Scatter Data Interpolation

This section details the derivation of sufficient conditions for *C* ^1^ triangular patches to be positive. Let the given positive scattered data set arranged over a triangular domain be {(*x*_*i*_, *y*_*i*_, *F*_*i*_), *i* = 1,2, …, *n*}. The resulting surface *S*(*x*, *y*) described as

$$\begin{array}{c} S\left(x_{i},y_{i}\right)=F_{i},i=1,2,...,n, \end{array}$$ (5)

is positive if

$$\begin{array}{c} S(x,y)>0,\forall (x,y)\in D. \end{array}$$ (6)

### 4.1 Domain Triangulation

Triangulation of data is performed by Delaunay triangulation method such that data *F*_*i*_, fall on vertices {*V*_*i*_ = (*x*_*i*_, *y*_*i*_), *i* = 1,2,3, …, *n*} of the triangles.

### 4.2 Estimation of Derivatives

Partial derivatives at the vertices *V*_*i*_, *i* = 1,2,3 of each triangle are calculated by derivative estimation scheme suggested by Goodman et. al. [15]

### 4.3 *C* ^1^ Positive triangular patch

**Fig 1:**
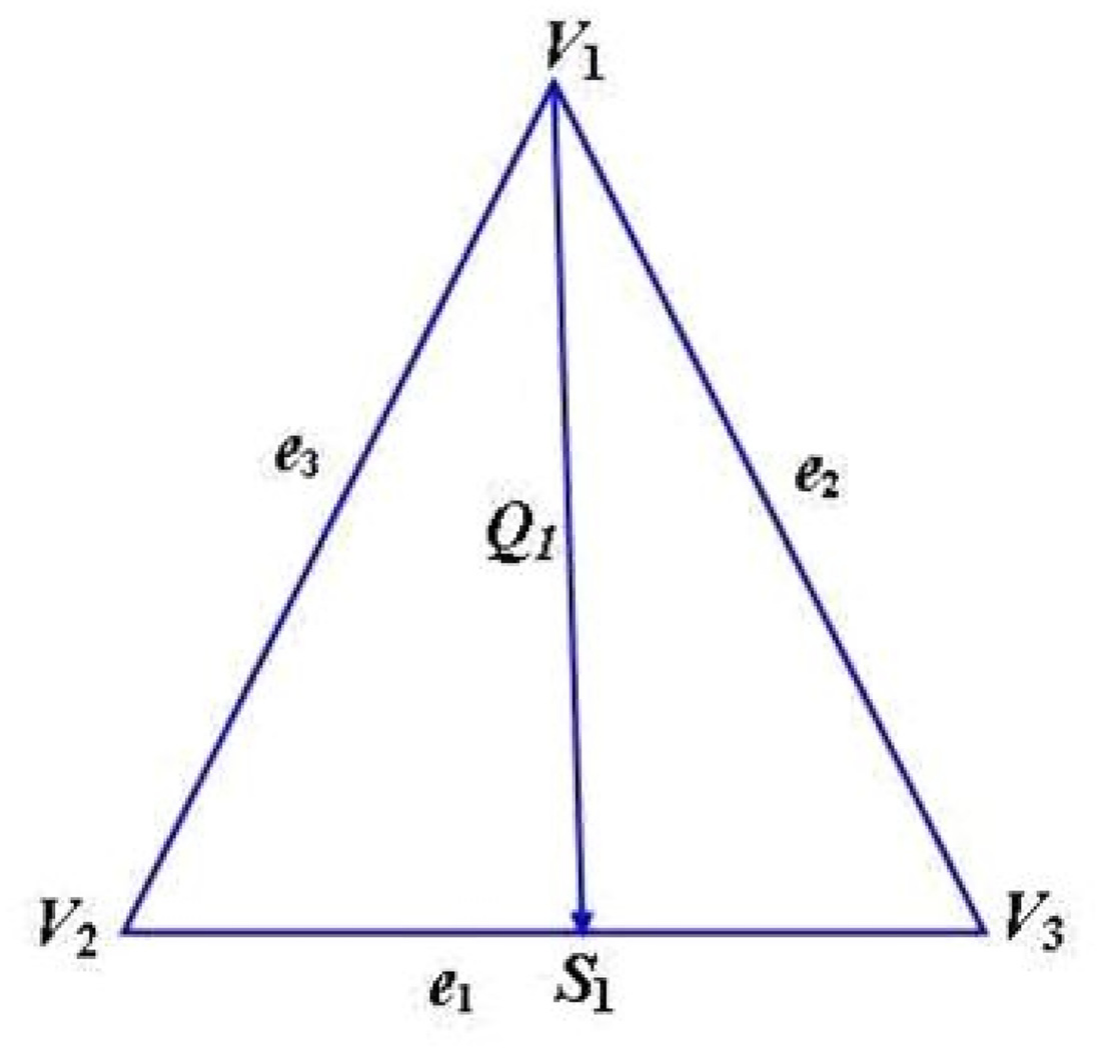
Radial curve *Q*_1_: connecting vertex *V*_1_ to the point *S*_1_.

Let *V*_1_ *V*_2_ *V*_3_ be the given triangle with edges *e*_*i*_, *i* = 1,2,3 opposite to the vertices *V*_*i*_, *i* = 1,2,3 respectively and *S*_*i*_, *i* = 1,2,3 be the points on the edges opposite to vertices *V*_*i*_, *i* = 1,2,3. The radial curve *Q*_1_ connecting vertex *V*_1_ to the points *S*_1_ on the opposite edges *e*_1_ is defined as (Fig 1):

$$\begin{array}{c} Q_{1}=\frac{Q_{1n}}{Q_{1d}}, \end{array}$$ (7)

where

$$\begin{array}{ccc} Q_{1n} & = & {\left(1-\sin\lambda \right)}^{3}F_{1}\alpha_{1}+\sin\lambda {\left(1-\sin\lambda \right)}^{2}\left(\beta_{1}F_{1}+\frac{2R_{1}\alpha_{1}}{\pi }\right)\\ & + & \cos\hat{\lambda }{\left(1-\cos\hat{\lambda }\right)}^{2}\left(\gamma_{1}F\left(S_{1}\right)-\frac{2\delta_{1}R_{2}}{\pi }\right)+{\left(1-\cos\hat{\lambda }\right)}^{3}\delta_{1}F\left(S_{1}\right),\\ Q_{1d} & = & \alpha_{1}{\left(1-\sin\lambda \right)}^{3}+\beta_{1}\sin\lambda {\left(1-\sin\lambda \right)}^{2}+\gamma_{1}\cos\hat{\lambda }{\left(1-\cos\hat{\lambda }\right)}^{2}+\delta_{1}{\left(1-\cos\hat{\lambda }\right)}^{3}. \end{array}$$

such that

$$\begin{array}{c} \lambda =\frac{\pi }{2}\left(1-u\right),\hat{\lambda }=1-\lambda . \end{array}$$

**Fig 2:**
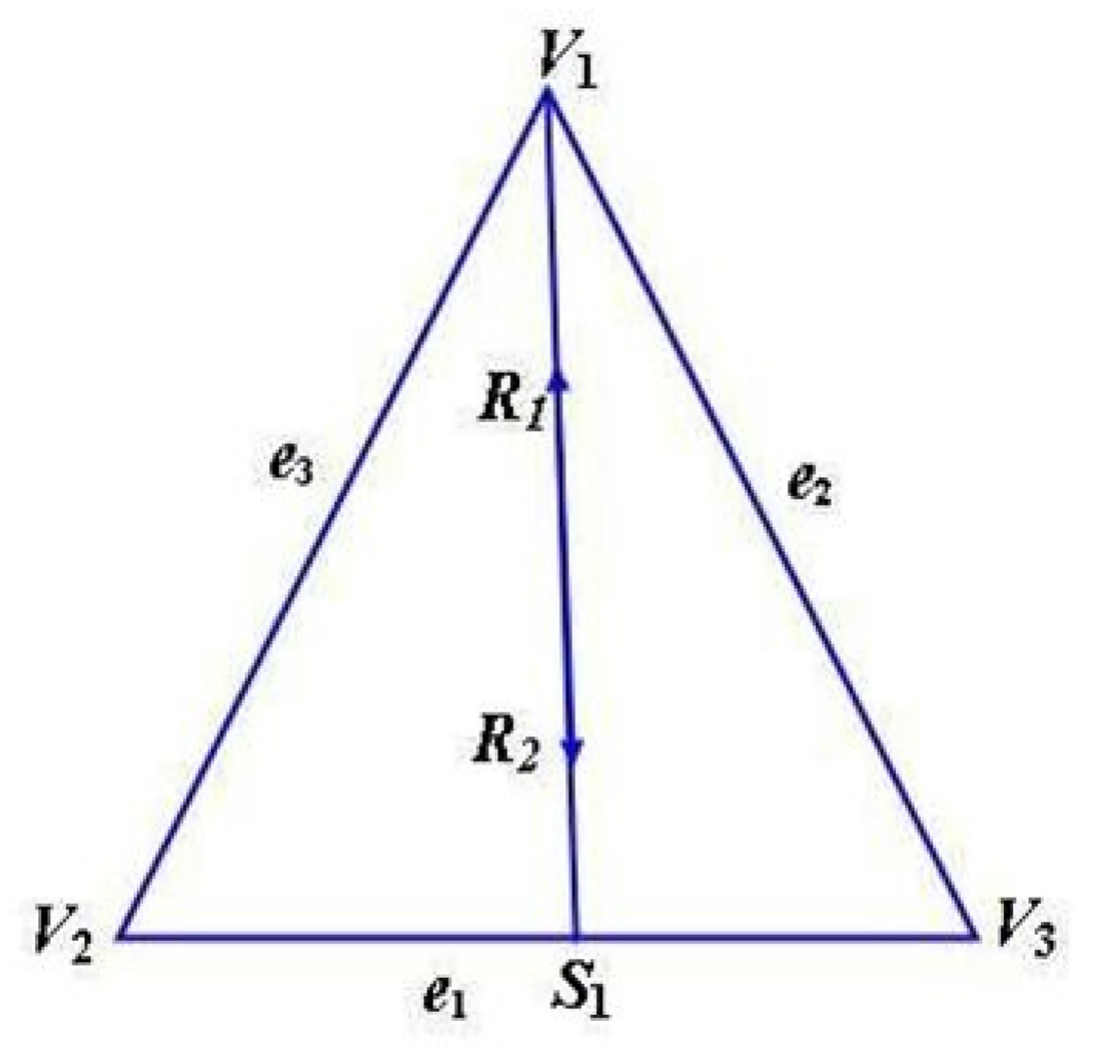
Directional derivatives along $\vec{S_{1}V_{1}}$ .

*R*_1_ and *R*_2_ are the directional derivatives at *V*_1_ and *S*_1_ (Fig 2) defined as

$$\begin{array}{ccc} R_{1} & = & \left(x_{s_{1}}-x_{1}\right)\frac{\partial f}{\partial x}\left(V_{1}\right)+\left(y_{s_{1}}-y_{1}\right)\frac{\partial f}{\partial y}\left(V_{1}\right),\\ R_{2} & = & \left(x_{s_{1}}-x_{1}\right)\frac{\partial f}{\partial x}\left(S_{1}\right)+\left(y_{s_{1}}-y_{1}\right)\frac{\partial f}{\partial y}\left(S_{1}\right). \end{array}$$

and *F*(*S*_1_) is the boundary curve along the edge *e*_1_ evaluated from the following expression

$$F(S_{1})=\frac{F_{1n}}{F_{1d}},$$

where

$$\begin{array}{ccc} F_{1n} & = & {\left(1-\sin r\right)}^{3}F_{2}\alpha_{4}+\sin r{\left(1-\sin r\right)}^{2}\left(\beta_{4}F_{2}+\frac{2d_{3}\alpha_{4}}{\pi }\right)\\ & + & \cos\hat{r}{\left(1-\cos\hat{r}\right)}^{2}\left(\gamma_{4}F_{3}-\frac{2\delta_{4}d_{4}}{\pi }\right)+{\left(1-\cos\hat{r}\right)}^{3}\delta_{4}F_{3},\\ F_{1d} & = & \alpha_{4}{\left(1-\sin r\right)}^{3}+\beta_{4}\sin r{\left(1-\sin r\right)}^{2}+\gamma_{4}\cos\hat{r}{\left(1-\cos\hat{r}\right)}^{2}+\delta_{4}{\left(1-\cos\hat{r}\right)}^{3}. \end{array}$$

such that

$$\begin{array}{c} r=\frac{\pi (1-v)}{2(v+w)},\hat{r}=\frac{\pi (1-w)}{2(u+w)}. \end{array}$$

**Fig 3:**
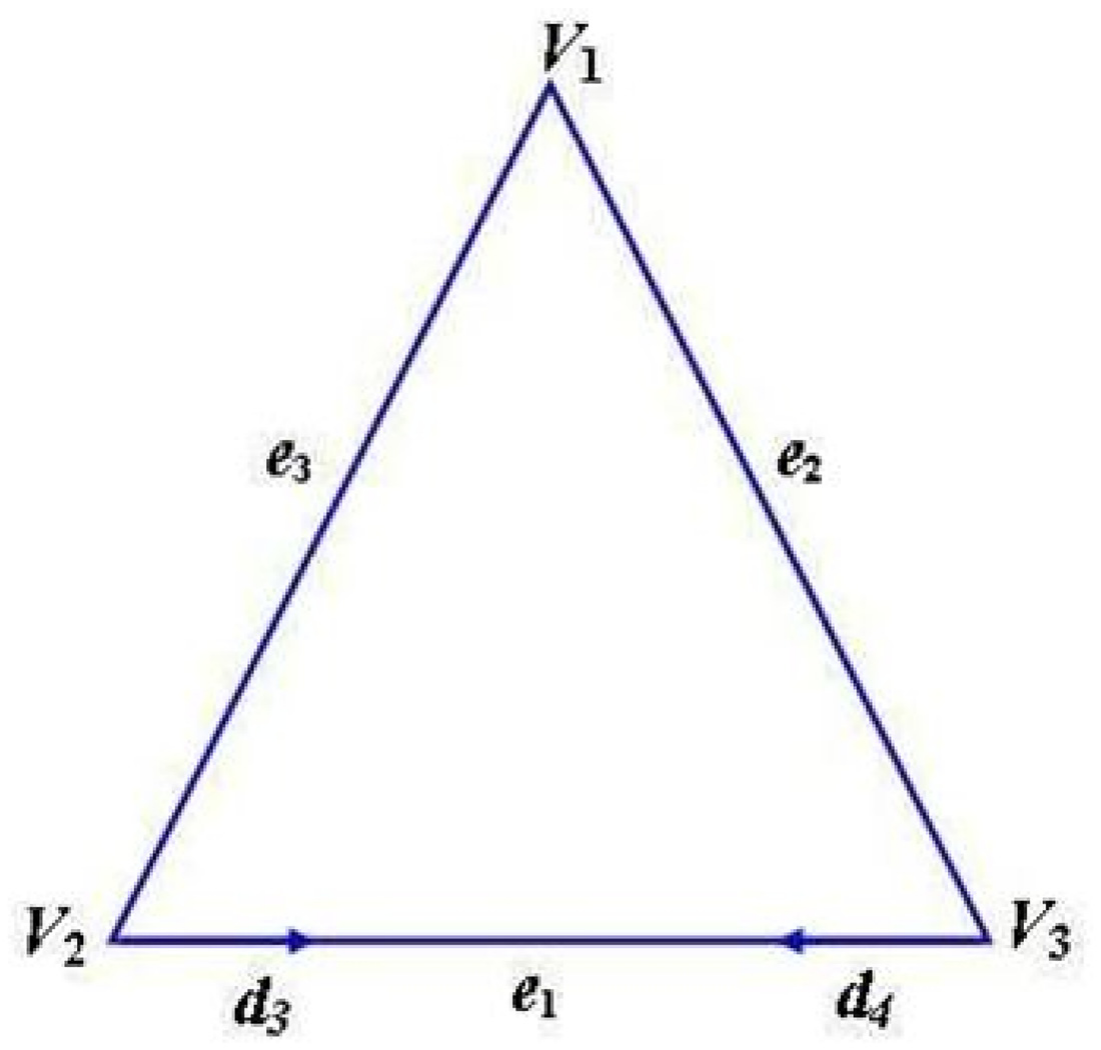
Directional derivatives along $\vec{V_{2}V_{3}}$.

*d*_3_ and *d*_4_ are the directional derivatives along $\vec{V_{2}V_{3}}$ at *V*_2_ and *V*_3_ (Fig 3)

$$\begin{array}{c} d_{3}=\left(x_{3}-x_{2}\right)\frac{\partial f}{\partial x}\left(V_{2}\right)+\left(y_{3}-y_{2}\right)\frac{\partial f}{\partial y}\left(V_{2}\right),\\ d_{4}=\left(x_{3}-x_{2}\right)\frac{\partial f}{\partial x}\left(V_{3}\right)+\left(y_{3}-y_{2}\right)\frac{\partial f}{\partial y}\left(V_{3}\right). \end{array}$$

From Eq 7, *Q*_1_ > 0 if

$$\begin{array}{c} Q_{1n}>0\text{and}Q_{1d}>0. \end{array}$$

Now, *Q*_1*n*_ > 0 if

$$\begin{array}{c} \alpha_{1}>0,\;\delta_{1}>0,\\ \beta_{1}>\frac{-2R_{1}\alpha_{1}}{\pi F_{1}},\\ \gamma_{1}>\frac{2R_{2}\delta_{1}}{\pi F(S_{1})},\\ F(S_{1})>0\;\;\;. \end{array}$$ (8)

From Theorem 2.1, *F*(*S*_1_) > 0 if

$$\begin{array}{c} \beta_{4}>\frac{-2\alpha_{4}d_{3}}{\pi F_{2}},\gamma_{4}>\frac{2\delta_{4}d_{4}}{\pi F_{3}}. \end{array}$$ (9)

Now, *Q*_1*d*_ > 0 if

$$\begin{array}{c} \alpha_{1}>0,\beta_{1}>0,\gamma_{1}>0\;\text{and}\;\delta_{1}>0. \end{array}$$

Likewise, radial curve *Q*_2_ connecting vertex *V*_2_ to the points *S*_2_ on the opposite edges *e*_2_ is defined as

$$\begin{array}{c} Q_{2}=\frac{Q_{2n}}{Q_{2d}}, \end{array}$$ (10)

where

$$\begin{array}{ccc} Q_{2n} & = & {\left(1-\sin\mu \right)}^{3}F_{2}\alpha_{2}+\sin\mu {\left(1-\sin\mu \right)}^{2}\left(\beta_{2}F_{2}+\frac{2R_{3}\alpha_{2}}{\pi }\right)\\ & + & \cos\hat{\mu }{\left(1-\cos\hat{\mu }\right)}^{2}\left(\gamma_{2}F\left(S_{2}\right)-\frac{2\delta_{2}R_{4}}{\pi }\right)+{\left(1-\cos\hat{\mu }\right)}^{3}\delta_{2}F\left(S_{2}\right),\\ Q_{2d} & = & \alpha_{2}{\left(1-\sin\mu \right)}^{3}+\beta_{2}\sin\mu {\left(1-\sin\mu \right)}^{2}+\gamma_{2}\cos\hat{\mu }{\left(1-\cos\hat{\mu }\right)}^{2}+\delta_{2}{\left(1-\cos\hat{\mu }\right)}^{3}. \end{array}$$

such that

$$\mu =\frac{\pi }{2}\left(1-v\right),\hat{\mu }=1-\mu .$$ (11)

*R*_3_ and *R*_4_ are the directional derivatives at *V*_2_ and *S*_2_

$$\begin{array}{ccc} R_{3} & = & \left(x_{s_{2}}-x_{2}\right)\frac{\partial f}{\partial x}\left(V_{2}\right)+\left(y_{s_{2}}-y_{2}\right)\frac{\partial f}{\partial y}\left(V_{2}\right)\\ R_{4} & = & \left(x_{s_{2}}-x_{2}\right)\frac{\partial f}{\partial x}\left(S_{2}\right)+\left(y_{s_{2}}-y_{2}\right)\frac{\partial f}{\partial y}\left(S_{2}\right) \end{array}$$

and *F*(*S*_2_) is the boundary curve along the edge *e*_2_ to be evaluated from the following expression

$$F(S_{2})=\frac{F_{2n}}{F_{2d}},$$

where

$$\begin{array}{ccc} F_{2n} & = & {\left(1-\sin s\right)}^{3}F_{3}\alpha_{5}+\sin r{\left(1-\sin r\right)}^{2}\left(\beta_{5}F_{3}+\frac{2d_{5}\alpha_{5}}{\pi }\right)\\ & + & \cos r{\left(1-\cos r\right)}^{2}\left(\gamma_{5}F_{1}-\frac{2\delta_{5}d_{6}}{\pi }\right)+{\left(1-\cos r\right)}^{3}\delta_{5}F_{1},\\ F_{2d} & = & \alpha_{5}{\left(1-\sin r\right)}^{3}+\beta_{5}\sin r{\left(1-\sin r\right)}^{2}+\gamma_{5}\cos r{\left(1-\cos r\right)}^{2}+\delta_{5}{\left(1-\cos r\right)}^{3}. \end{array}$$

*d*_5_ and *d*_6_ are the directional derivatives along $\vec{V_{1}V_{3}}$ at *V*_1_ and *V*_3_

$$\begin{array}{ccc} d_{5} & = & \left(x_{1}-x_{3}\right)\frac{\partial f}{\partial x}\left(V_{3}\right)+\left(y_{1}-y_{3}\right)\frac{\partial f}{\partial y}\left(V_{3}\right),\\ d_{6} & = & \left(x_{3}-x_{2}\right)\frac{\partial f}{\partial x}\left(V_{1}\right)+\left(y_{3}-y_{2}\right)\frac{\partial f}{\partial y}\left(V_{1}\right). \end{array}$$

From Eq 10, *Q*_2_ > 0 if

$$\begin{array}{c} Q_{2n}>0\text{and}Q_{2d}>0. \end{array}$$

Now, *Q*_2*n*_ > 0 if

$$\begin{array}{c} \alpha_{2}>0,\;\delta_{2}>0,\\ \beta_{2}>\frac{-2R_{3}\alpha_{2}}{\pi F_{2}},\\ \gamma_{2}>\frac{2R_{4}\delta_{2}}{\pi F(S_{2})},\\ F(S_{2})>0\;\;\;. \end{array}$$ (12)

From Theorem 2.1, *F*(*S*_2_) > 0 if

$$\begin{array}{c} \beta_{5}>\frac{-2\alpha_{5}d_{5}}{\pi F_{3}},\gamma_{5}>\frac{2\delta_{5}d_{6}}{\pi F_{1}}. \end{array}$$ (13)

Now, *Q*_2*d*_ > 0 if

$$\begin{array}{c} \alpha_{2}>0,\beta_{2}>0,\gamma_{2}>0\;\text{and}\;\delta_{2}>0. \end{array}$$

and the radial curve *Q*_3_ connecting vertex *V*_3_ to the point *S*_3_ on the opposite edge *e*_3_ is defined as

$$\begin{array}{c} Q_{3}=\frac{Q_{3n}}{Q_{3d}}, \end{array}$$ (14)

where

$$\begin{array}{ccc} Q_{3n} & = & {\left(1-\sin\nu \right)}^{3}F_{3}\alpha_{3}+\sin\nu {\left(1-\sin\nu \right)}^{2}\left(\beta_{3}F_{3}+\frac{2R_{5}\alpha_{3}}{\pi }\right)\\ & + & \;\cos\nu {\left(1-\cos\nu \right)}^{2}\left(\gamma_{3}F\left(S_{3}\right)-\frac{2\delta_{3}R_{6}}{\pi }\right)+{\left(1-\cos\nu \right)}^{3}\delta_{3}F\left(S_{3}\right),\\ Q_{3d} & = & \alpha_{3}{\left(1-\sin\nu \right)}^{3}+\beta_{3}\sin\nu {\left(1-\sin\nu \right)}^{2}+\gamma_{3}\cos\nu {\left(1-\cos\nu \right)}^{2}+\delta_{3}{\left(1-\cos\nu \right)}^{3}. \end{array}$$

where *R*_5_ and *R*_6_ are the directional derivatives at *V*_3_ and *S*_3_ defined as

$$\begin{array}{ccc} R_{5} & = & \left(x_{s_{3}}-x_{3}\right)\frac{\partial f}{\partial x}\left(V_{3}\right)+\left(y_{s_{3}}-y_{3}\right)\frac{\partial f}{\partial y}\left(V_{3}\right)\\ R_{6} & = & \left(x_{s_{3}}-x_{3}\right)\frac{\partial f}{\partial x}\left(S_{3}\right)+\left(y_{s_{3}}-y_{3}\right)\frac{\partial f}{\partial y}\left(S_{3}\right) \end{array}$$

and *F*(*S*_3_) is the boundary curve along the edge *e*_3_ to be evaluated from the following expression

$$F(S_{3})=\frac{F_{3n}}{F_{3d}},$$

where

$$\begin{array}{ccc} F_{3n} & = & {\left(1-\sin t\right)}^{3}F_{1}\alpha_{6}+\sin t{\left(1-\sin t\right)}^{2}\left(\beta_{6}F_{1}+\frac{2d_{1}\alpha_{6}}{\pi }\right)\\ & + & \cos t{\left(1-\cos t\right)}^{2}\left(\gamma_{6}F_{2}-\frac{2\delta_{6}d_{2}}{\pi }\right)+{\left(1-\cos t\right)}^{3}\delta_{6}F_{2},\\ F_{3d} & = & \alpha_{6}{\left(1-\sin t\right)}^{3}+\beta_{6}\sin t{\left(1-\sin t\right)}^{2}+\gamma_{6}\cos t{\left(1-\cos t\right)}^{2}+\delta_{6}{\left(1-\cos t\right)}^{3}. \end{array}$$

*d*_1_ and *d*_2_ are the directional derivatives along $\vec{V_{1}V_{2}}$ at *V*_1_ and *V*_2_

$$\begin{array}{ccc} d_{5} & = & \left(x_{2}-x_{1}\right)\frac{\partial f}{\partial x}\left(V_{1}\right)+\left(y_{2}-y_{1}\right)\frac{\partial f}{\partial y}\left(V_{1}\right),\\ d_{6} & = & \left(x_{2}-x_{1}\right)\frac{\partial f}{\partial x}\left(V_{2}\right)+\left(y_{2}-y_{1}\right)\frac{\partial f}{\partial y}\left(V_{2}\right). \end{array}$$

From Eq 14, *Q*_3_ > 0 if

$$\begin{array}{c} Q_{3n}>0\;\text{and}\;Q_{3d}>0. \end{array}$$

Now, *Q*_3*n*_ > 0 if

$$\begin{array}{c} \alpha_{3}>0,\;\delta_{2}>0,\\ \beta_{3}>\frac{-2R_{5}\alpha_{3}}{\pi F_{3}},\\ \gamma_{3}>\frac{2R_{6}\delta_{3}}{\pi F(S_{3})},\\ F(S_{3})>0\;\;\;. \end{array}$$ (15)

From Theorem 2.1, *F*(*S*_3_) > 0 if

$$\beta_{6}>\frac{-2\alpha_{6}d_{1}}{\pi F_{1}},\gamma_{6}>\frac{2\delta_{6}d_{2}}{\pi F_{2}}.$$ (16)

Now, *Q*_3*d*_ > 0 if

$$\begin{array}{c} \alpha_{3}>0,\beta_{3}>0,\gamma_{3}>0\;\text{and}\;\delta_{3}>0. \end{array}$$

The above discussion leads to the following result:

**Theorem 4.1** *The C^1^ triangular pactch P in* Eq (4) *is positive if the following conditions are attained.*

$$\begin{array}{c} \alpha_{1}>0,\alpha_{2}>0,\alpha_{3}>0,\alpha_{4}>0,\alpha_{5}>0,\alpha_{6}>0,\\ \delta_{1}>0,\delta_{2}>0,\delta_{3}>0,\delta_{4}>0,\delta_{5}>0,\delta_{6}>0,\\ \beta_{1}>\max\left\{0,\frac{-2R_{1}\alpha_{1}}{\pi F_{1}}\right\},\gamma_{1}>\max\left\{0,\frac{2R_{2}\delta_{1}}{\pi F(S_{1})}\right\},\\ \beta_{2}>\max\left\{0,\frac{-2R_{3}\alpha_{2}}{\pi F_{2}}\right\},\gamma_{2}>\max\left\{0,\frac{2R_{4}\delta_{2}}{\pi F(S_{2})}\right\},\\ \beta_{3}>\max\left\{0,\frac{-2R_{5}\alpha_{3}}{\pi F_{3}}\right\},\gamma_{3}>\max\left\{0,\frac{2R_{6}\delta_{3}}{\pi F(S_{3})}\right\},\\ \beta_{4}>\max\left\{0,\frac{-2\alpha_{4}d_{3}}{\pi F_{2}}\right\},\gamma_{4}>\max\left\{0,\frac{2\delta_{4}d_{4}}{\pi F_{3}}\right\},\\ \beta_{5}>\max\left\{0,\frac{-2\alpha_{5}d_{5}}{\pi F_{3}}\right\},\gamma_{5}>\max\left\{0,\frac{2\delta_{5}d_{6}}{\pi F_{1}}\right\},\\ \beta_{6}>\max\left\{0,\frac{-2\alpha_{6}d_{1}}{\pi F_{1}}\right\},\gamma_{6}>\max\left\{0,\frac{2\delta_{6}d_{2}}{\pi F_{2}}\right\}. \end{array}$$

*The above constraints can be rearranged as*

$$\begin{array}{c} \beta_{1}>l_{1}+\max\left\{0,\frac{-2R_{1}\alpha_{1}}{\pi F_{1}}\right\},\gamma_{1}>m_{1}+\max\left\{0,\frac{2R_{2}\delta_{1}}{\pi F(S_{1})}\right\};l_{1},m_{1}>0,\\ \beta_{2}>l_{2}+\max\left\{0,\frac{-2R_{3}\alpha_{2}}{\pi F_{2}}\right\},\gamma_{2}>m_{2}+\max\left\{0,\frac{2R_{4}\delta_{2}}{\pi F(S_{2})}\right\};l_{2},m_{2}>0,\\ \beta_{3}>l_{3}+\max\left\{0,\frac{-2R_{5}\alpha_{3}}{\pi F_{3}}\right\},\gamma_{3}>m_{3}+\max\left\{0,\frac{2R_{6}\delta_{3}}{\pi F(S_{3})}\right\};l_{3},m_{3}>0,\\ \beta_{4}>l_{4}+\max\left\{0,\frac{-2\alpha_{4}d_{3}}{\pi F_{2}}\right\},\gamma_{4}>m_{4}+\max\left\{0,\frac{2\delta_{4}d_{4}}{\pi F_{3}}\right\};l_{4},m_{4}>0,\\ \beta_{5}>l_{5}+\max\left\{0,\frac{-2\alpha_{5}d_{5}}{\pi F_{3}}\right\},\gamma_{5}>m_{5}+\max\left\{0,\frac{2\delta_{5}d_{6}}{\pi F_{1}}\right\};l_{5},m_{5}>0,\\ \beta_{6}>l_{6}+\max\left\{0,\frac{-2\alpha_{6}d_{1}}{\pi F_{1}}\right\},\gamma_{6}>m_{6}+\max\left\{0,\frac{2\delta_{6}d_{2}}{\pi F_{2}}\right\};l_{6},m_{6}>0. \end{array}$$

## 5. Numerical Examples

This section illustrates the positivity preserving scheme for scattered data devised in Section 4.3.

**Fig 4:**
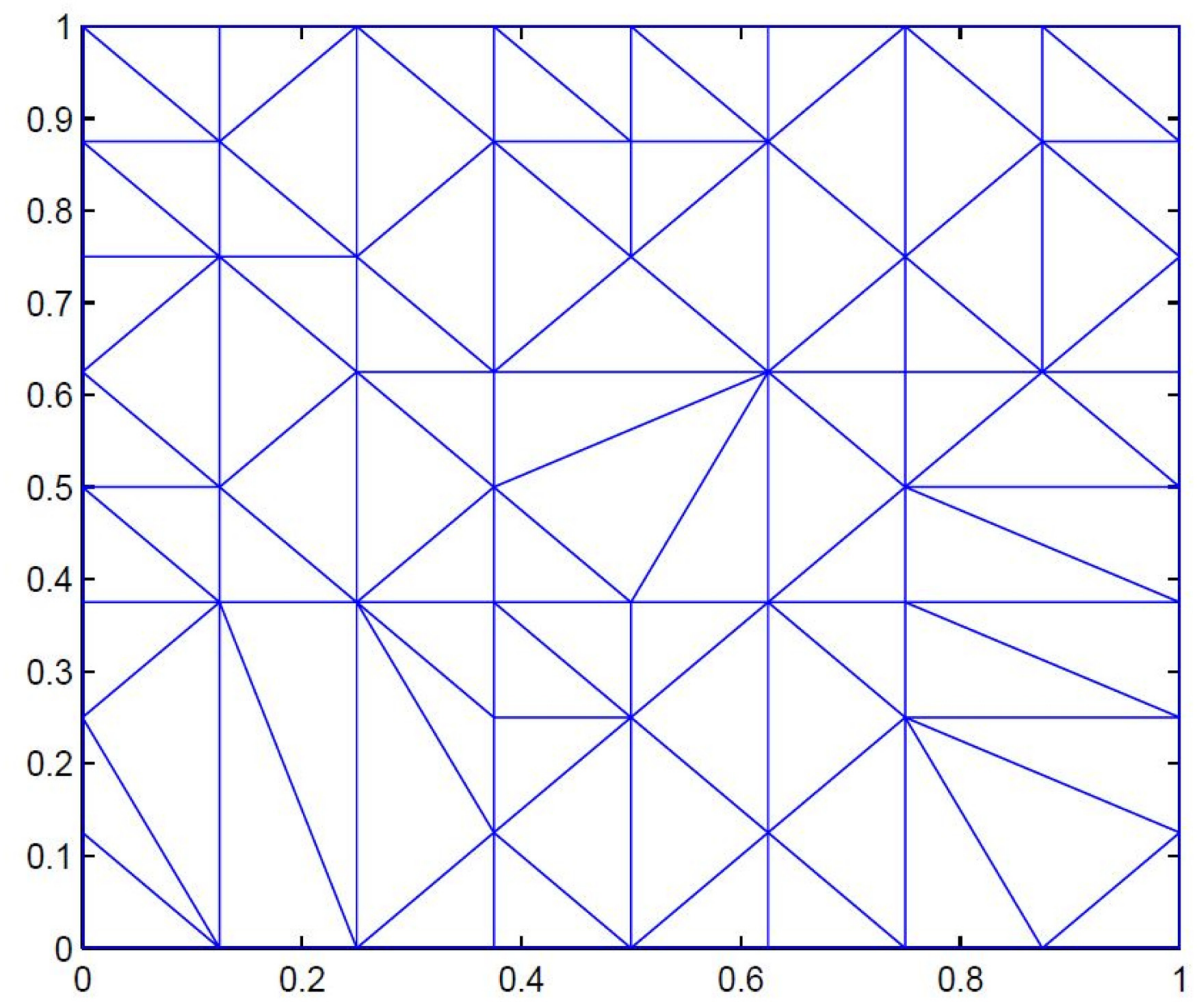
Delaunay triangulation of positive data in Table 1.

**Fig 5:**
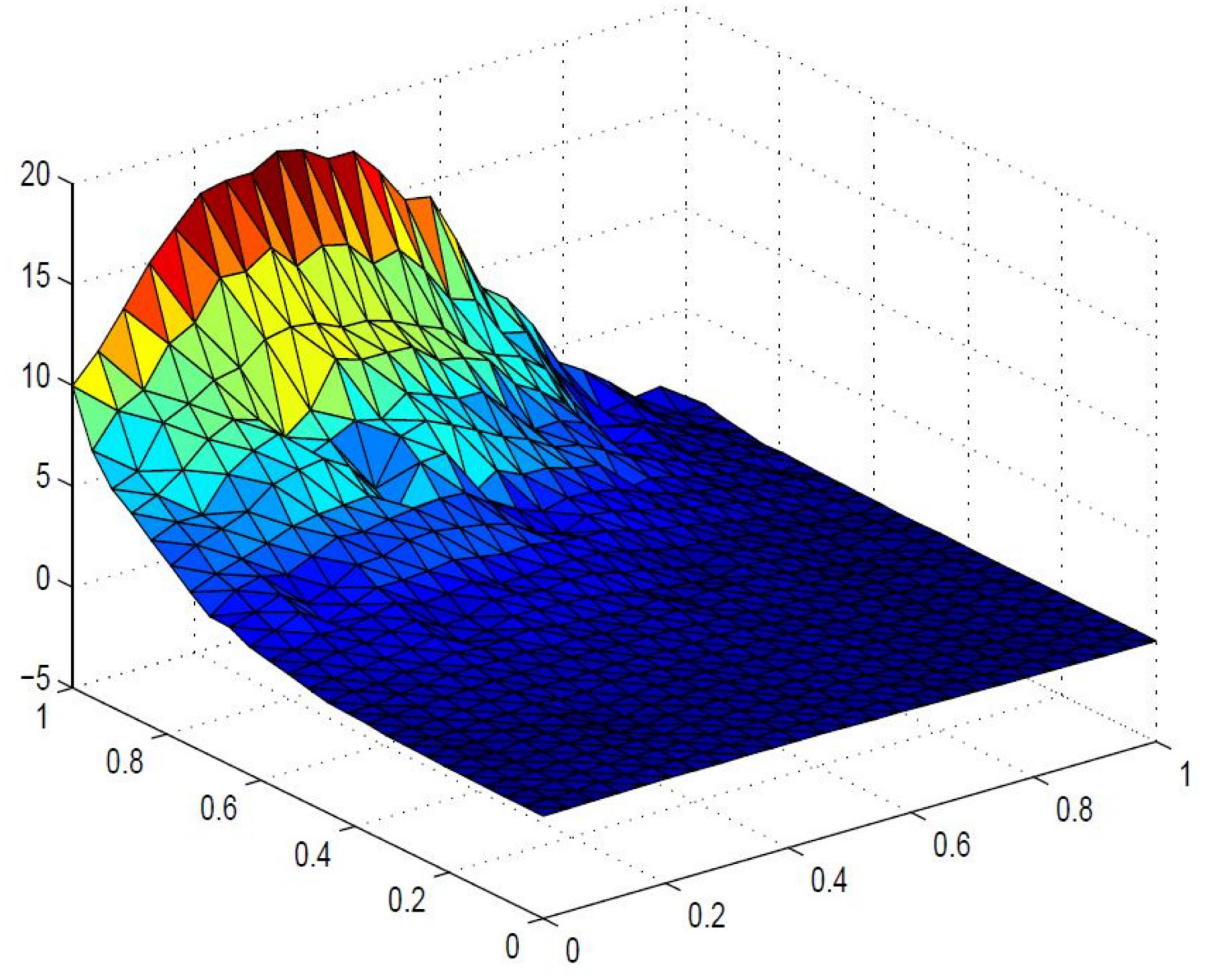
Rational cubic trigonometric surface of the positive data in Table 1.

**Fig 6:**
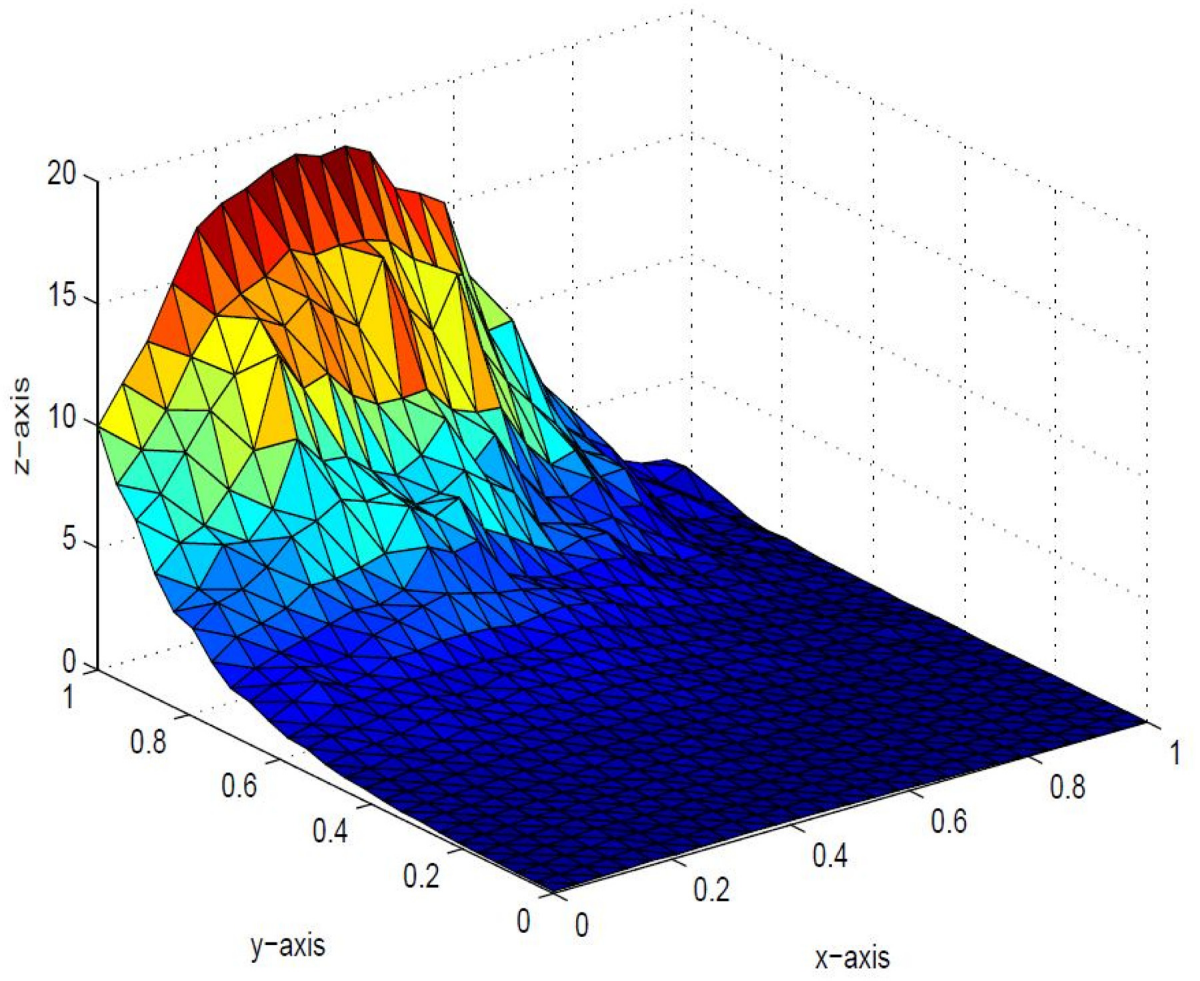
Positive surface generated from Theorem 4.1 of the positive data in Table 1.

**Fig 7:**
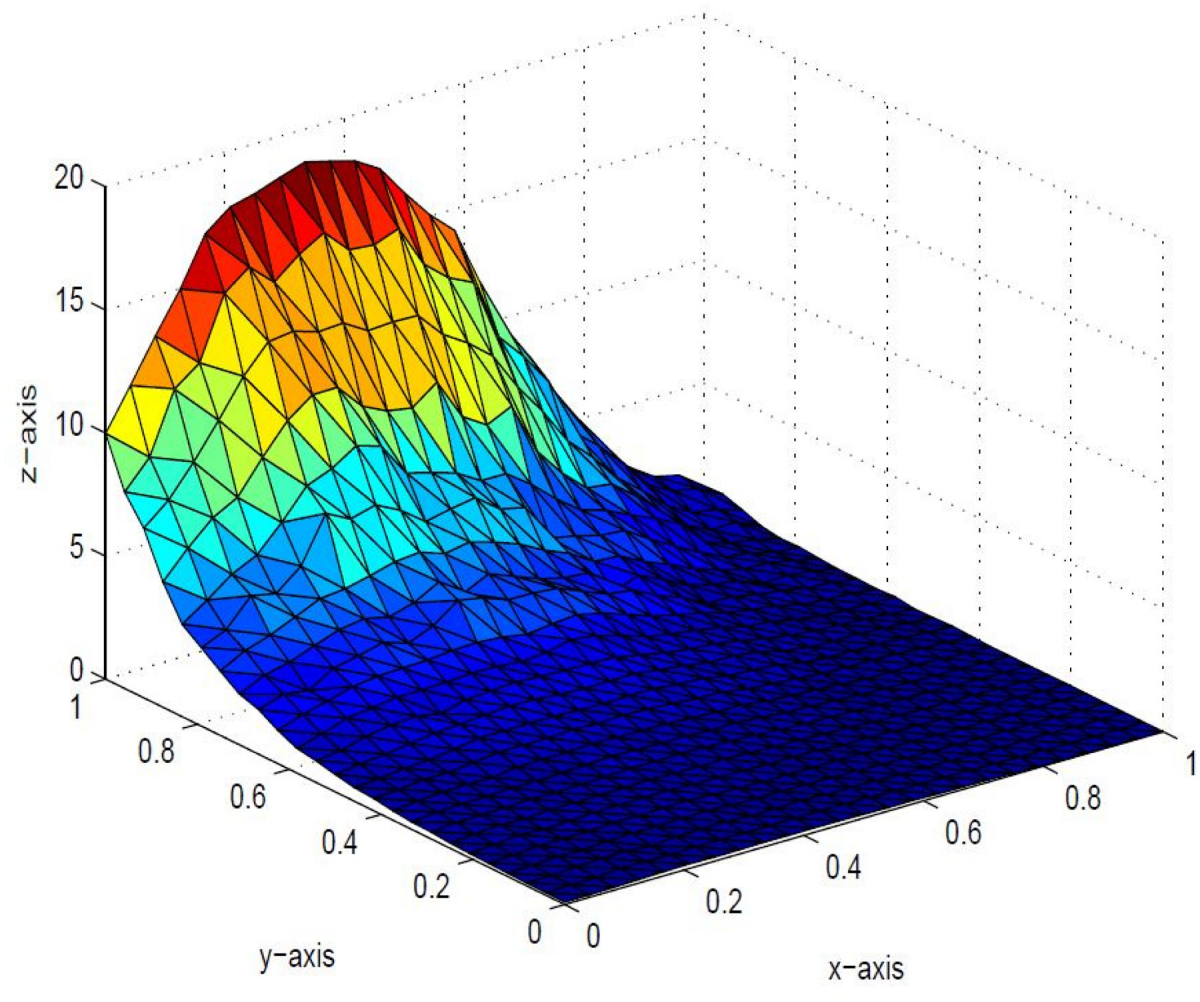
Positive surface generated from Theorem 4.1 of the positive data in Table 1.

**Fig 8:**
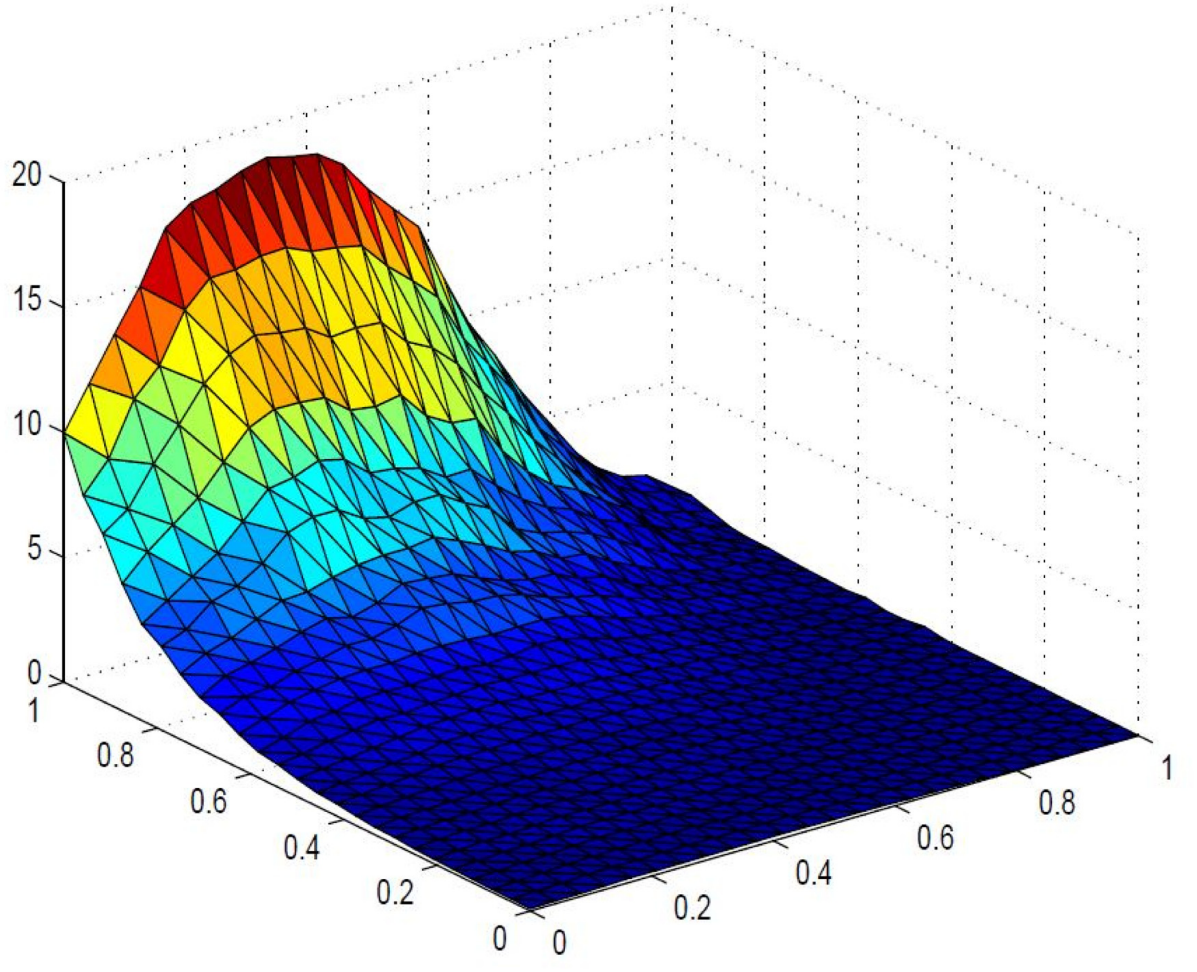
Positive surface generated from Theorem 4.1 of the positive data in Table 1.

**Example 5.1** *Positive scattered data is taken in* Table 1. Fig 4 *represents corresponding delaunay triangulations. The data is interpolated first by* Eq (4) *for arbitrary values of free parameters*, *α*_1_ = 4.1, *α*_2_ = 3;*α*_3_ = 2.5, *α*_4_ = 1.6, *α*_5_ = 2.7, *α*_6_ = 2.8, *β*_1_ = 3.8, *β*_2_ = 2.4, *β*_3_ = 4.2, *β*_4_ = 2.5, *β*_5_ = 1.5, *β*_6_ = 4, *γ*_1_ = 1, *γ*_2_ = 6, *γ*_3_ = 1, *γ*_4_ = 2, *γ*_5_ = 2, *γ*_6_ = 3, *δ*_1_ = 1, *δ*_2_ = 3, *δ*_3_ = 3, *δ*_4_ = 1, *δ*_5_ = 2, *δ*_6_ = 1. *The resulting surface is displayed in* Fig 5. *It is clear from* Fig 5 *that the inherent shape feature of positivity of data could no be held in visual model. This detriment is removed in* Figs 6, 7 and 8 *by implementing positivity preserving conditions summarized in Theorem 4.1. It is worth mentioning here that parameters* *α*_*i*_ *and* *δ*_*i*_ *for* *i* = 1,2, …,6 *are left free to refine the shape according to user’s requirement. The effect of free parameters are shown in* Figs 6, 7 and 8. Figs 6 and 7 *are constructed against the parameter choice* *α*_1_ = 12, *α*_2_ = 0.4, *α*_3_ = 13, *α*_4_ = 0.22, *α*_5_ = 12, *α*_6_ = 0.33, *δ*_1_ = 13, *δ*_2_ = 0.3, *δ*_3_ = 14, *δ*_4_ = 0.3, *δ*_5_ = 15, *δ*_6_ = 12 and *α*_1_ = 0.1, *α*_2_ = 1.0, *α*_3_ = 0.5, *α*_4_ = 1.6, *α*_5_ = 0.7, *α*_6_ = 0.8, *δ*_1_ = 0.8, *δ*_2_ = 0.4, *δ*_3_ = 1.2, *δ*_4_ = 1.5, *δ*_5_ = 1.5, *δ*_6_ = 1.0 *respectively, which lacks smoothness. A smooth visibly pleasant representation is obtained in* Fig 8 *by setting* *α*_1_ = 1.0, *α*_2_ = 1.0, *α*_3_ = 0.5, *α*_4_ = 0.6, *α*_5_ = 0.7, *α*_6_ = 0.8, *δ*_1_ = 0.8, *δ*_2_ = 0.4, *δ*_3_ = 1.2, *δ*_4_ = 0.5, *δ*_5_ = 1.0, *δ*_6_ = 1.0

**Table 1:** A Positive scattered data set I.

**x**	**y**	**F**
0	0	0.7487
0	0.125	0.5779
0	0.25	0.4668
0	0.375	0.4042
0	0.625	0.4042
0	0.75	0.4668
0	0.875	0.5779
0	1	0.7487
0.125	0	0.5779
0.125	0.125	0.4248
0.125	0.5	0.251
0.125	0.625	0.2691
0.125	0.75	0.3252
0.125	1	0.5779
0.25	0	0.4668
0.25	0.125	0.3252
0.25	0.25	0.2331
0.25	0.375	0.1813
0.25	0.5	0.1645
0.25	0.875	0.3252
0.375	0.125	0.2691
0.375	0.25	0.1813
0.375	0.625	0.1317
0.375	0.75	0.1813
0.375	0.875	0.2691
0.375	1	0.4042
0.5	0	0.384
0.5	0.375	0.1157
0.5	0.625	0.1157
0.5	0.75	0.1645
0.5	0.875	0.251
0.5	1	0.384
0.625	0	0.4042
0.625	0.125	0.2691
0.625	0.375	0.1317
0.625	0.5	0.1157
0.625	0.625	0.1317
0.75	0	0.4668
0.75	0.125	0.3252
0.75	0.375	0.1813
0.75	0.75	0.2331
0.75	0.875	0.3252
x	y	z
0.875	0	0.5779
0.875	0.125	0.4248
0.875	0.375	0.2691
0.875	0.625	0.2691
0.875	0.75	0.3252
0.875	0.875	0.4248
0.875	1	0.5779
1	0	0.7487
1	0.125	0.5779
1	0.25	0.4668
1	0.375	0.4042
1	0.5	0.384
1	0.625	0.4042
1	0.75	0.4668
1	0.875	0.5779
1	1	0.7487
0.75	1	0.4668

**Fig 9:**
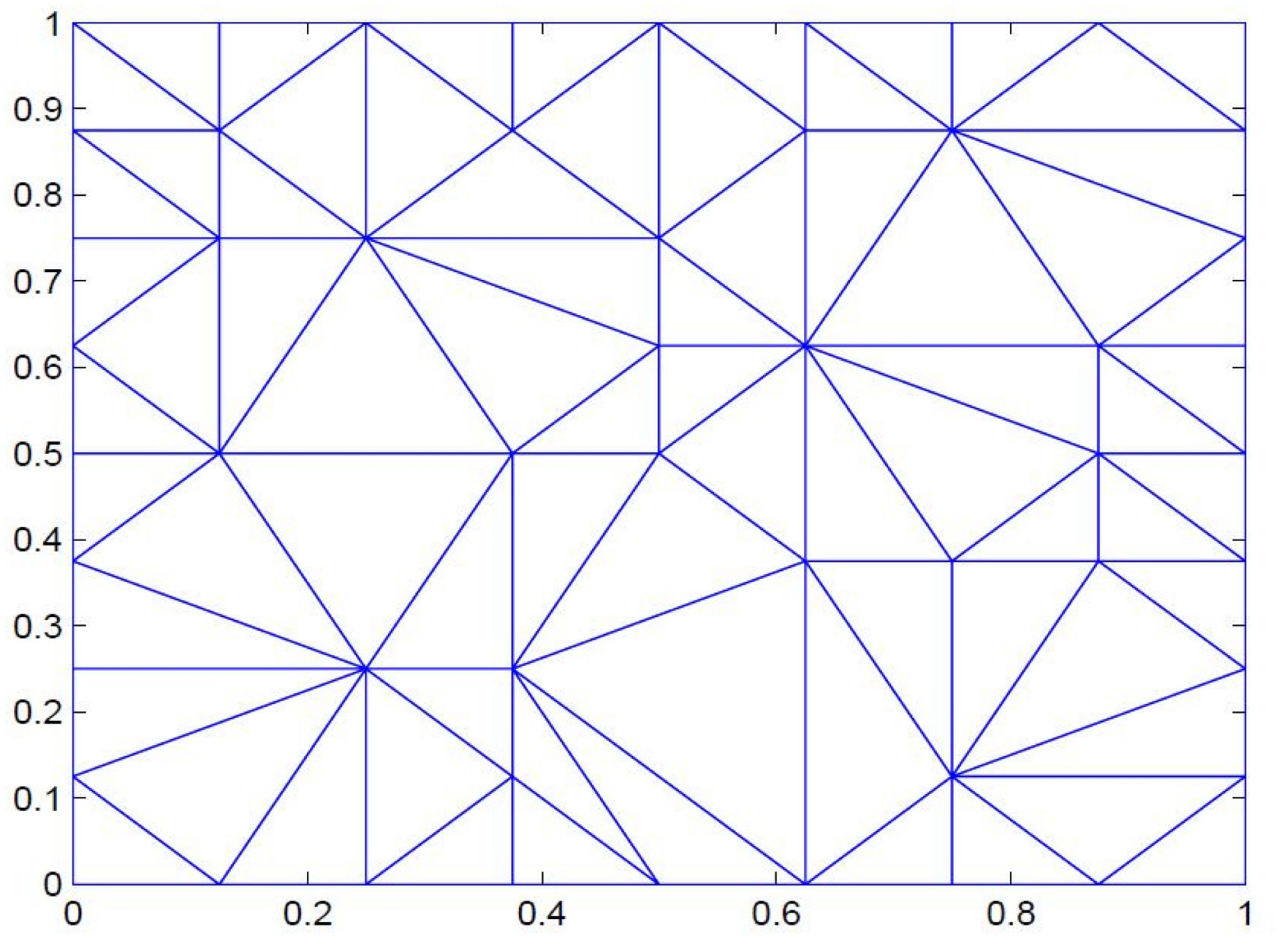
Delaunay triangulation of positive data in Table 2.

**Fig 10:**
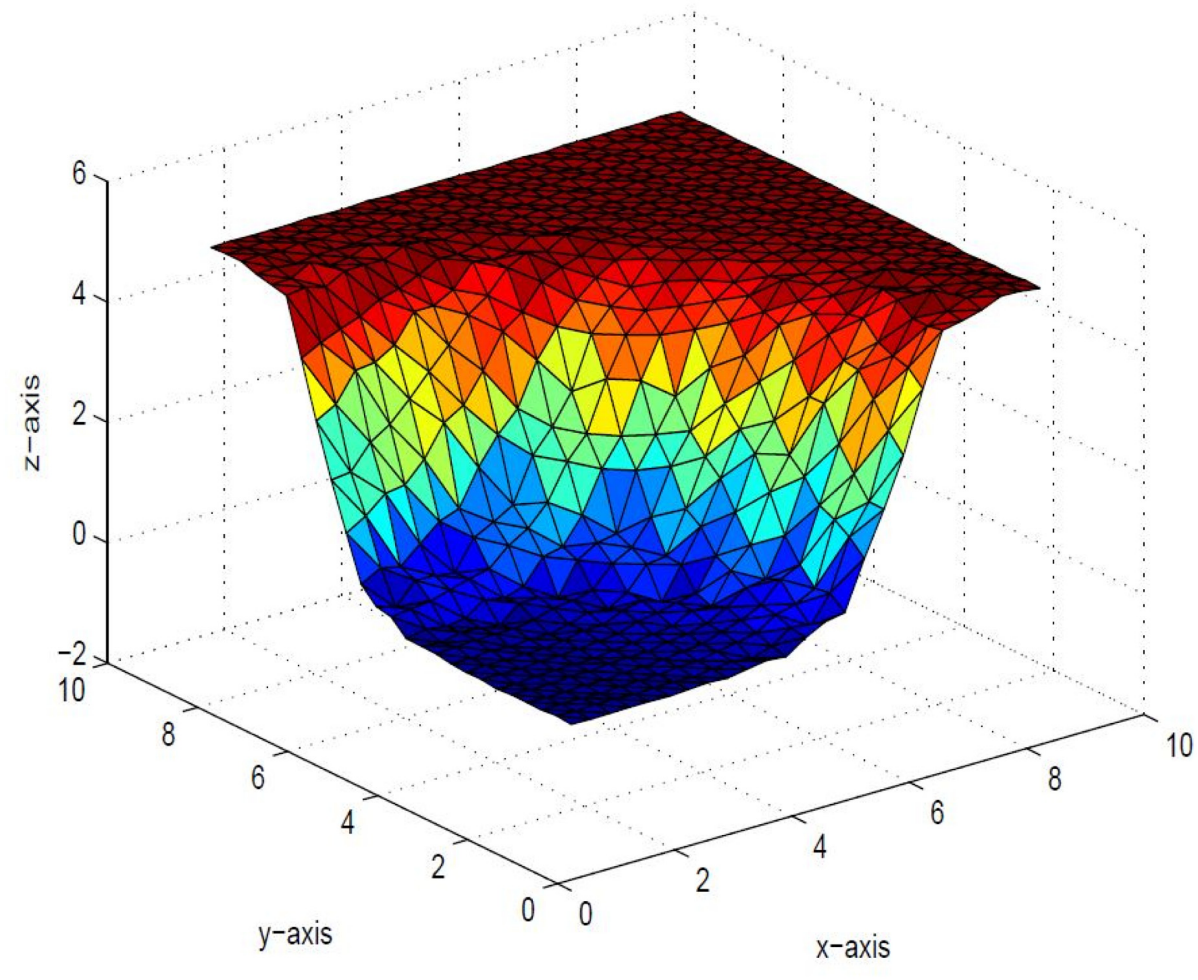
Rational cubic trigonometric surface of the positive data.

**Fig 11:**
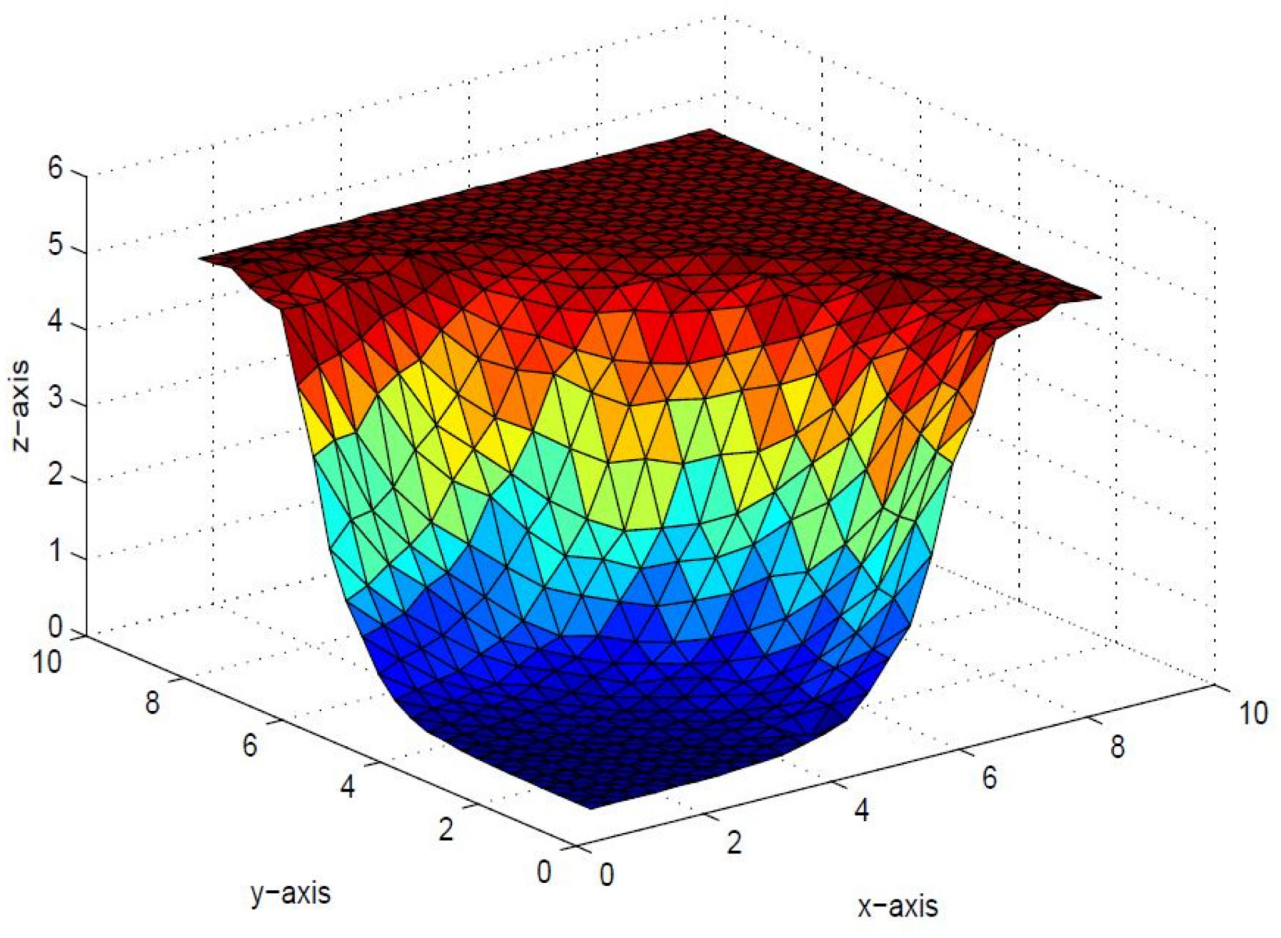
Positive surface generated from Theorem 4.1.

**Fig 12:**
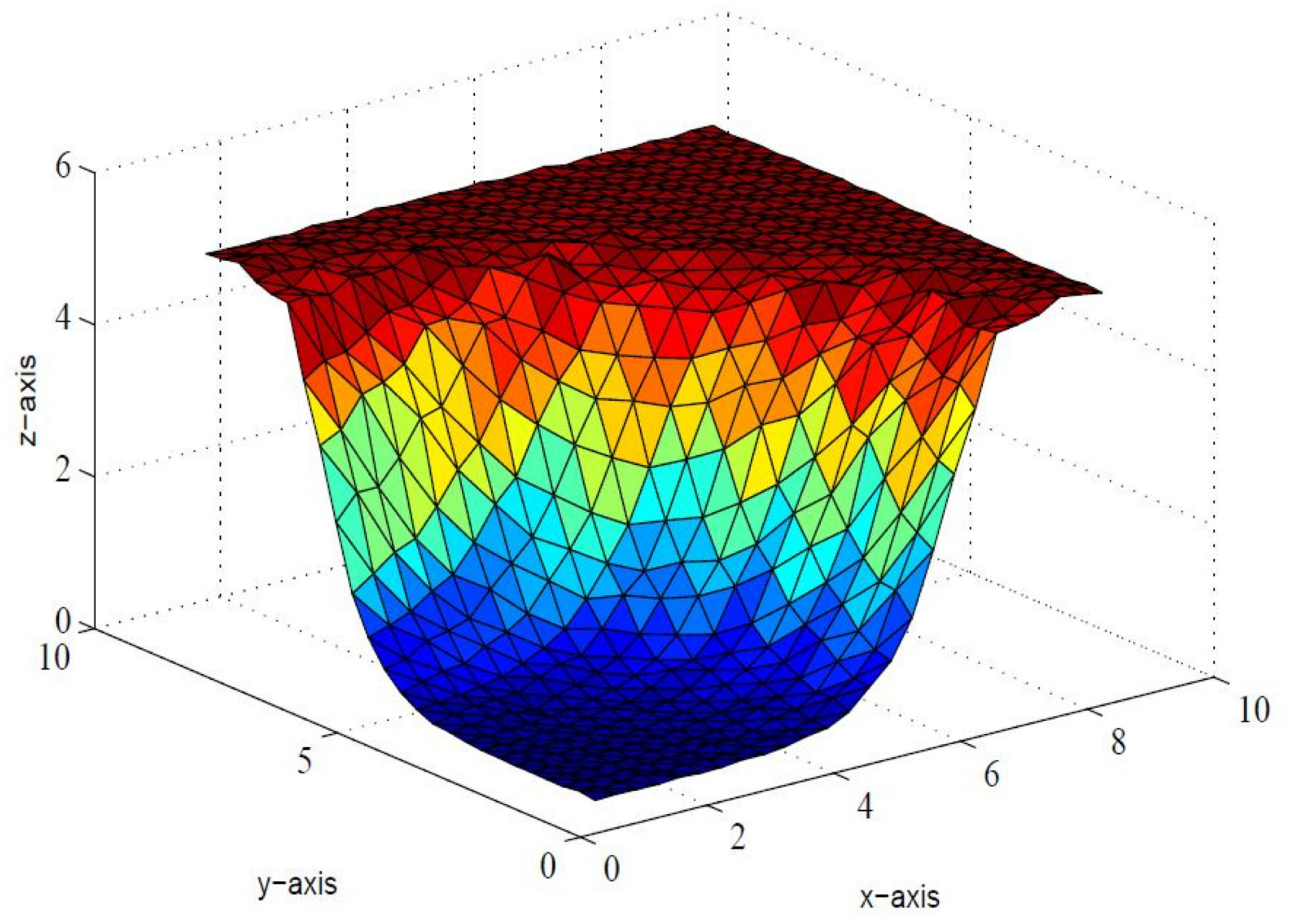
Positive surface generated from Theorem 4.1.

**Fig 13:**
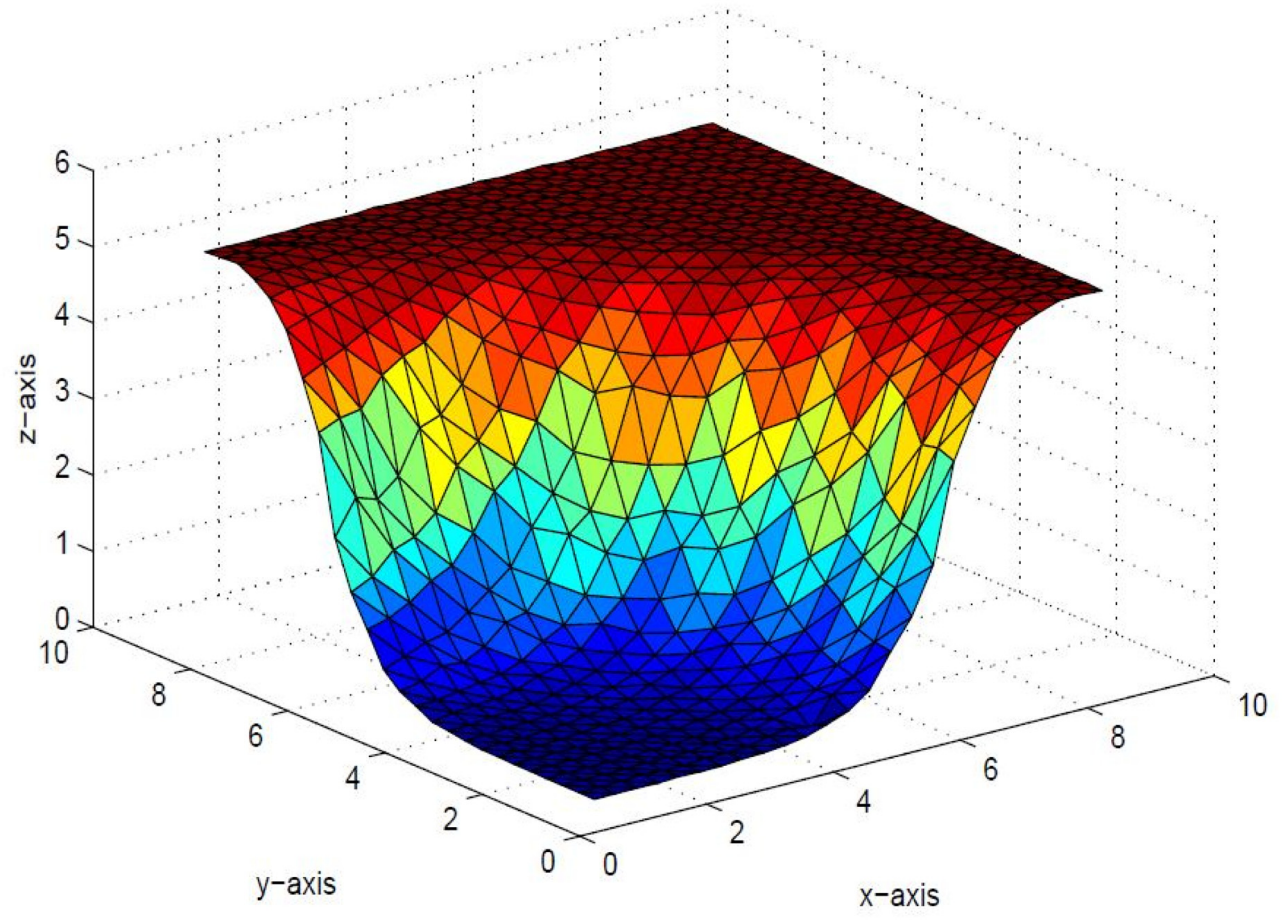
Positive surface generated from Theorem 4.1.

**Example 5.2** *A Positive scattered data set is displayed in* Table 2. *Delauny triangulation is illustrated in* Fig 9 *and the corresponding surface in* Fig 10 *is obtained by interpolating the data for arbitrary values of free parameters,* *α*_1_ = 4.1, *α*_2_ = 3;*α*_3_ = 2.0, *α*_4_ = 1.5, *α*_5_ = 2.7, *α*_6_ = 2.5, *β*_1_ = 3.5, *β*_2_ = 2.3, *β*_3_ = 3.2, *β*_4_ = 2.2, *β*_5_ = 1.0, *β*_6_ = 4.5, *γ*_1_ = 1.4, *γ*_2_ = 5.5, *γ*_3_ = 1.5, *γ*_4_ = 2.2, *γ*_5_ = 1.5, *γ*_1_ = 2.5, *δ*_1_ = 0.5, *δ*_2_ = 3.1, *δ*_3_ = 3.5, *δ*_4_ = 0.4, *δ*_5_ = 2, *δ*_6_ = 1.2, *in description of* Eq (4). *It is evident from* Fig 10 *that the positivity of data could not be conserved in visual model. This impediment is removed in* Figs 11, 12 *and* Fig 13 *by implementing positivity preserving constraints on parameters* *β*_*i*_, *γ*_*i*_ *for* *i* = 1,2, …,6, *summarized in Theorem 4.1. Here, it is noteworthy that parameters* *α*_*i*_ *and* *δ*_*i*_ *for* *i* = 1,2, …,6 *are set free to refine the shape as required by the user. The effect of free parameters are shown in* Figs 11, 12 and 13. Figs 11 *and* 12 *are constructed against the parameter choice* *α*_1_ = 2.0, *α*_2_ = 0.1, *α*_3_ = 0.5, *α*_4_ = 0.5, *α*_5_ = 1.0, *α*_6_ = 0.63, *δ*_1_ = 1.0, *δ*_2_ = 0.33, *δ*_3_ = 0.5, *δ*_4_ = 0.4, *δ*_5_ = 1.0, *δ*_6_ = 0.3 *and* *α*_1_ = 2.2, *α*_2_ = 1.1, *α*_3_ = 2.5, *α*_4_ = 1.5, *α*_5_ = 1.0, *α*_6_ = 1.0, *δ*_1_ = 1.0, *δ*_2_ = 1.0, *δ*_3_ = 1.5, *δ*_4_ = 1.4, *δ*_5_ = 1.0, *δ*_6_ = 0.3 *respectively, which lacks smoothness. A smooth visibly pleasant representation is obtained in* Fig 13 *by setting* *α*_1_ = 2.0, *α*_2_ = 0.4, *α*_3_ = 0.5, *α*_4_ = 0.5, *α*_5_ = 1.0, *α*_6_ = 0.63, *δ*_1_ = 0.3, *δ*_2_ = 0.33, *δ*_3_ = 0.5, *δ*_4_ = 0.3, *δ*_5_ = 0.5, *δ*_6_ = 0.2.

**Table 2:** A Positive scattered data set II.

**x**	**y**	**F**
0	0	0.4486
0	0.125	0.3616
0	0.25	0.4692
0	0.375	0.6827
0	0.5	0.786
0	0.625	0.836
0	0.75	0.8765
0	0.875	0.9125
0	1	0.9447
0.125	0	0.3369
0.125	0.125	0.0001
0.125	0.375	0.6256
0.125	0.625	0.8621
0.125	0.875	0.9334
0.125	1	0.9634
0.25	0	0.4529
0.25	0.125	0.1767
0.25	0.25	0.3217
0.25	0.375	0.7005
0.25	0.5	0.8555
0.25	0.625	0.9327
0.25	0.75	0.9775
0.25	0.875	0.9686
0.25	1	0.9926
0.375	0	0.696
0.375	0.375	0.8363
0.375	0.625	1.2176
0.375	0.875	1.028
0.375	1	1.0284
0.5	0	0.8329
0.5	0.125	0.8315
0.5	0.25	0.821
0.5	0.375	0.8498
0.5	0.5	0.925
0.5	0.625	1.0925
0.5	0.75	1.1688
0.5	0.875	1.0568
0.5	1	1.0662
0.625	0	0.9049
0.625	0.125	0.8376
0.625	0.375	0.7163
0.625	0.5	0.8608
0.625	0.75	1.0671
0.625	0.875	1.0883
0.625	1	1.1023
0.75	0	0.9639
0.75	0.125	0.8326
0.75	0.25	0.6283
0.75	0.375	0.5976
0.75	0.5	0.8075
0.75	0.625	1.0136
0.75	0.75	1.0989
0.75	0.875	1.1231
0.75	1	1.134
0.875	0	1.0355
0.875	0.125	0.922
0.875	0.25	0.7477
0.875	0.375	0.7193
0.875	0.5	0.893
0.875	0.625	1.0638
0.875	0.75	1.1335
0.875	0.875	1.152
0.875	1	1.1597
1	0	1.1074
1	0.125	1.0598
1	0.25	0.9848
1	0.375	0.9745
1	0.5	1.054
1	0.625	1.1319
1	0.75	1.1646
1	0.875	1.1744
1	1	1.1791

## Conclusion

In this study, positivity preserving algorithm for scattered data arranged over a triangular domain, is established. The rational trigonometric cubic function [13] with four free parameters is used for the interpolation along each boundary and radial curve. Nielson side vertex has been applied to construct the interpolating surface. Constraints on half of the parameters are obtained to guarantee the positive shape of data while half are set free for users modification. The proposed algorithm, surpasses many prevailing approaches in literature. In [10], authors utilized a cubic function with one free parameter to retain the positive shape of data. Positive surface was obtained by drawing data dependent constraints on this free parameter, and, hence the scheme did not offer refinement in the shape. The scheme suggested in this paper does not suffer this detriment. The developed algorithm is local and can be applied to data with or without derivatives. Moreover, shape preserving algorithms play an instrumental role in many areas of visualization such as geometric modelling, robot trajectories, evolution game theory, prisoner’s dilemma game [16], [17], [18], meshless method and inverse kinemaics etc.
